# Establishing stereotactic body radiotherapy with flattening filter free techniques in the treatment of pulmonary lesions - initial experiences from a single institution

**DOI:** 10.1186/s13014-016-0648-0

**Published:** 2016-06-08

**Authors:** Juliane Rieber, Eric Tonndorf-Martini, Oliver Schramm, Bernhard Rhein, Laila König, Sebastian Adeberg, Eva Meyerhof, Angela Mohr, Jutta Kappes, Hans Hoffmann, Jürgen Debus, Stefan Rieken

**Affiliations:** Department of Radiation Oncology, University Hospital Heidelberg, Im Neuenheimer Feld 400, 69120 Heidelberg, Germany; Heidelberg Institute of Radiation Oncology, Im Neuenheimer Feld 400, 69120 Heidelberg, Germany; Translational Research Unit, Thoraxklinik, Heidelberg University, Germany Translational Lung Research Centre Heidelberg (TLRC-H), Member of the German Centre for Lung Research (DZL), Heidelberg, Germany; Department of Pneumology, Thoraxklinik, Heidelberg University, Heidelberg, Germany; Department of Thoracic Surgery, Thoraxklinik, Heidelberg University, Heidelberg, Germany

## Abstract

**Background:**

Stereotactic body radiotherapy (SBRT) using flattening filter free (FFF)-techniques has been increasingly applied during the last years. However, clinical studies investigating this emerging technique are still rare. Hence, we analyzed toxicity and clinical outcome of pulmonary SBRT with FFF-techniques and performed dosimetric comparison to conventional techniques using flattening filters (FF).

**Materials and methods:**

Between 05/2014 and 06/2015, 56 consecutive patients with 61 pulmonary lesions were treated with SBRT in FFF-mode. Central lesions received 8 × 7.5 Gy delivered to the conformally enclosing 80 %-isodose, while peripheral lesions were treated with 3 × 15 Gy, prescribed to the 65 %-isodose. Early and late toxicity (after 6 months) as well as initial clinical outcomes were evaluated. Furthermore, [deleted] plan quality and efficiency were evaluated by analyzing conformity, beam- on and total treatment delivery times in comparison to plans with FF-dose application.

**Results:**

Median follow-up time was 9.3 months (range 1.5–18.0 months). Early toxicity was low with only 5 patients (8.9 %) reporting CTCAE 2° or higher side-effects. Only one patient (1.8 %) was diagnosed with radiation-induced pneumonitis CTCAE 3°, while 2 (3.6 %) patients suffered from pneumonitis CTCAE 2°. After 6 months, no toxicity greater than CTCAE 2° was reported. 1-year local progression-free survival, distant progression-free survival and overall survival were 92.8 %, 78.0 %, and 94.4 %, respectively. While plan quality was similar for FFF- and FF-plans in respect to conformity (*p* = 0.275), median beam-on time as well as total treatment time were significantly reduced for SBRT in FFF-mode compared to FF-mode (*p* ≤ 0.001, *p* ≤ 0.001).

**Conclusions:**

Patient treatment with SBRT using FFF-techniques is safe and provides promising clinical results with only modest toxicity at significantly increased dose delivery speed.

## Background

About 20 % of all patients diagnosed with early-stage NSCLC or with oligometastasized tumors to the lung are classified medically inoperable due to cardiopulmonary comorbidities resulting in insufficient pulmonary function [1–3]. Stereotactic body radiotherapy (SBRT) represents the treatment of choice for these patients who often only received best supportive care in former years. Indeed, several recent studies about SBRT treatment for both, early stage NSCLC and pulmonary metastases, showed excellent local control rates of 78–96 %, which is considerably better compared to historical data applying conventional radiotherapy [4–9]. Interestingly, a recent study of two pooled randomized trials even suggested that SBRT treatment might even be an option for medically operable patients diagnosed with stage I NSCLC [10]. For oligometastastic patients, SBRT is discussed as a cytoreductive treatment option during systemic therapy [11, 12]. Hence, SBRT is an optimal technique as it offers the possibility of target dose escalation for maximizing tumor control, while sparing surrounding lung tissue for minimizing toxicity.

However, SBRT delivery times often last long due to high doses per fraction, limited dose rates, application of multiple treatment beams and often usage of intensity-modulated radiotherapy (IMRT) for further improving dose conformality [13]. On the one hand, duration of SBRT treatment is stressful for a frail elderly patient population frequently suffering from severe cardiopulmonary comorbidities and who are commonly positioned within rigid and uncomfortable full body casts. On the other hand, extended treatment times might increase the risk of tumor displacement due to intrafractional patient movement possibly leading to extra imaging for position verification [14].

For accelerating delivery, the dose rate of a beam can be increased by the removal of the flattening filter (FF) resulting in a cone-shaped dose profile and up to a fourfold higher dose rate in the center of the beam [15]. Furthermore, using these flattening filter free (FFF)-techniques a reduction of the out-of field dose is expected possibly leading to decreased exposure to normal tissue [16, 17].

In Heidelberg, SBRT using FFF-techniques has been clinically available for more than one year. This report describes planning procedures and analyzes toxicity and oncological outcome in 61 patients with pulmonary lesions treated in our department.

## Materials and methods

### Patients and pulmonary lesions

Patient characteristics are summarized in Table 1. Between May 2014 and June 2015, 56 patients with 61 pulmonary lesions underwent SBRT using FFF-techniques at the Department of Radiooncology at the University Hospital in Heidelberg. All patients were diagnosed functionally inoperable due to severe [deleted] pulmonary comorbidities. Additionally, all patients underwent contrast-enhanced CT scans for diagnosis, while patients with limited stage NSCLC further received FDG-PET-CT scans. The analysis was approved by the Ethics committee of the University Hospital Heidelberg (S-140/2016). Written informed consent for publication of their images was obtained from the respective patients. [deleted]

**Table 1:** Patient characteristics of 56 patients with 61 pulmonary lesions

Patients	
Sex	
Male	37 (66.1 %)
Female	19 (33.9 %)
Median age (range)	70.4 years (42.7–87.0)
≥ 70 years	31 (55.4 %)
< 70 years	25 (44.6 %)
Baseline Karnofsky Index	
	70 % (50–80 %)
Pulmonary function (median)	
FEV_1_ (%)	62 % (27.8–120.4 %)
FEV_1_ (absolut)	1.7 l (0.83–4.38 l)
Smoking status	
Active smokers	25 (44.6 %)
Former smokers	20 (35.8 %)
Never-smokers	6 (10.7 %)
Smoking status not known	5 (8.9 %)
Median packyears	43 (20–120)
Pulmonary lesions	
TNM limited stage NSCLC patients	
cT1cN0	14
cT2cN0	14
cT3cN0	0
cT4cN0 (further metastases in other lobe)	2
TNM locally advanced NSCLC patients	
cT1cN1-3	4
cT2cN1-3	1
Pulmonary metastases – primary tumors	
NSCLC	19
Breast cancer	2
Renal cell carcinoma	2
Prostate cancer	1
Sarcoma	1
Cancer of unkown primary	1

### Planning and treatment features

Patients were immobilized in individually shaped body casts and an additional abdominal compression device was used on the upper abdomen for reduction of respiratory tumor motion of lesions in the lower lobes. For treatment planning, a thoracic CT scan with a slice thickness of 3 mm was performed with the patient in treatment position under shallow breathing. Additionally, to account for respiratory motion, a 4-dimensional CT scan during normal breathing was acquired.

Gross tumor volumes (GTV) were contoured on 3 mm CT slices in several maximal-extension phases (including maximal inspiration and expiration as well as the middle breathing position) from the 4D-planning CT and summed up to an internal target volume (ITV). To account for microscopic infiltration, a 5 mm safety margin was added to form the clinical target volume (CTV). An additional planning target volume (PTV) margin of 2 mm was further added to account for positioning insecurities. Central airway structures with a margin of 2 cm were contoured to identify lesions beyond (“peripheral”) or within (“central”) this structure and to determine the dose prescribed. Central lesions were treated with 8 × 7.5 Gy delivered to the conformally enclosing 80 %-isodose with desired dose maxima of up to 75 Gy. Peripheral lesions were planned to receive 3 × 15 Gy, prescribed to the 65 %-isodose with desired maximum doses of up to 69 Gy. Due to severe pulmonary comorbidities and hence no other treatment options, five patients with locally advanced NSCLC with a small, peripheral primary tumor were treated with SBRT to the primary tumor, while the local lymph node metastases received conventional radiotherapy (Table 1). These five patients additionally received intensity-modulated radiotherapy to the mediastinal and supraclavicular lymph node metastases with median total dose of 60.0 Gy (range 54.0–60.0 Gy) delivered in 27–30 fractions. Organs at risk (OARs) and normal tissue constraints were adopted as recently described by the Stereotactic Radiotherapy Working Group of the German Society of Radiation Oncology [18].

Delivery techniques comprised 3-D conventional (*n* = 30) and VMAT (*n* = 31) radiotherapy using the FFF-mode. Additional plans in FF-mode were calculated for all patients in the respective technique (3-D and VMAT). The same number of fields or arcs was used for the corresponding plans in FF-mode. For VMAT plans 1 arc (*n* = 18), 2 arcs (*n* = 11) and 3 arcs (*n* = 2) were used, while for 3-D-conventional plans 6 fields (*n* = 3); 7 fields (*n* = 12), 8 fields (*n* = 12) and 9 fields (*n* = 3) were applied. Plans were designed and calculated using Oncentra (version 4.5). [deleted] A Collapsed Cone (CC) algorithm was used for dose calculation. All patients were treated with 6 MV FFF-plans using the Elekta Versa HD with a maximum dose rate of 1400 MU/min and 6 MV FF-plans were calculated for comparison for each lesion. The modified Paddick Conformity Index was applied for comparison [19, 20]:

$$ CI=\frac{V_{ptv, pi}^2}{V_{ptv}{V}_{pi}}, $$

where *V*_*ptv,pi*_ is the partial volume of the PTV covered by the prescribed isodose, *V*_*ptv*_ is the planning target volume and *V*_*pi*_ is the body volume of the patient covered by the prescribed isodose. Hence, a score of 1.0 indicates perfect conformity, while a score of less than 1 shows worse conformity [19].

Beam-on time, as well as total treatment time, was registered for all patients during each day of treatment. Furthermore, beam-on and total treatment time were also calculated for FF-plans for comparison.

Before the initial irradiation, image guidance was performed by means of KV cone beam CT (CBCT). A position verification was furthermore applied before each fraction by orthogonal portal images being compared with digitally reconstructed radiographs (DRR) from the planning CT.

### Outcome evaluation

All patients were seen for follow-up visits at the University Hospital in Heidelberg and underwent a clinical examination and a CT or an X-ray scan of the thorax. The first follow-up examination was performed 6–8 weeks after radiotherapy with a CT scan and the following visits were scheduled every 3 months for the first two years and afterwards every 6 months. If no pathology was detected in the CT scan, CT scans and X-rays scans were done alternately every three months. Statistical comparisons were performed with SPSS (version 20.0) using the non-parametric Wilcoxon signed-rank test. Significance was noted for two-tailed *p*-values of ≤ 0.05. Outcome was calculated using the Kaplan-Meier-Method and treatment-related toxicity was classified according to CTCAE v4.0. Local control (LC) was defined as no progression of the tumor within the treated area. Recurrences distant to the primary pulmonary lesion in the same lobe were not classified as local failure but as distant metastases. Overall and progression-free survival as well as local and distant progression-free survival was analyzed starting from the end of radiotherapy. Furthermore, LC was calculated for all pulmonary lesions (61 pulmonary lesions), while OS, PFS and DPFS were analyzed for all patients (56 patients).

## Results

### Planning procedure and technical administration

In total, 61 pulmonary lesions were treated using SBRT in the FFF-mode. While 25 lesions were classified as central, 36 were peripheral lesions. [deleted] Mean PTV was 58.2 ml (range: 6.1–99.8 ml).

To show comparability between FFF- and FF-techniques, we generated both FFF- and FF-plans for all patients and subjected them to comparative physical verification. [deleted] The FFF-plans were non-inferior with a median conformity index of 0.71 (range 0.42–0.92), while the FF-plans showed a median conformity index of 0.69 (range 0.37–0.91) (*p* = 0.275).

As one advantage of SBRT treatment with the FFF-technique is believed to be accelerated dose delivery, we registered the treatment time per fraction starting from the acquisition of CBCT and portal images until the end of radiation for the 61 pulmonary lesions. While the median treatment time for the FFF-technique plans was found to be 8:17 min (range 5:02–13:54 min), treatment with FF-plans lasted significantly longer with a median time of 9:47 min (range 6:12–16:32 min) (*p* ≤ 0.001). Only focusing on beam-on time, treatment with FFF-plans was found to be more than two times faster in median compared to irradiation with FF-plans (1:29 vs. 3:24 min) (*p* ≤ 0.001).

### Toxicity

Detailed patient and tumor characteristics’ are shown in Table 1. All patients were classified functionally inoperable due to severe pulmonary disease. [deleted]

Table 2 shows mean doses for organs at risks (OARs): for peripheral lesions treated with 3 × 15 Gy prescribed to the 65 %-isodose and for central lesions treated with 8 × 7.5 Gy prescribed to the 80 %- isodose.

**Table 2:** Summary of dose volume histogram doses for organs at risks

Organs at risk	Peripheral lesions	Central lesions
Mean dose ipsilateral lung (Gy)	4.9	11.1
Mean dose contralateral lung (Gy)	0.9	2.2
Mean dose both lungs (Gy)	2.9	6.7
D_2%_-dose spinal cord (Gy)	6.9	12.5
D_2%-_dose central airways (Gy)	5.8	19.4
D_2%-_dose esophagus (Gy)	8.4	17.3

Dose in Gy shown; D_2%_: dose received by at least 2 % of the volume

Data for early toxicity was available for all patients. In general, early toxicity was low with only 5 patients (8.9 %) suffering from CTCAE 2° or higher side-effects (Table 3). Main acute side-effects were cough (25.0 %) and general disorders/ fatigue (12.5 %). Radiation-induced pneumonitis was diagnosed in 7 patients (12.5 %) after 3 months, while pneumonitis CTCAE 2° and 3° was only detected in one patient each during the first 3 months (Table 3). The patient diagnosed with radiation-induced pneumonitis CTCAE 3° had already suffered from an oxygen-dependent COPD (GOLD IV) before SBRT. Treatment comprised increased oxygen therapy and oral steroids which led to a permanent remission of the clinical and radiological symptoms and changes.

**Table 3:** observed acute side-effects classified according to Common Terminology Criteria Adverse Events, version 4.0

	CTCAE, version 4.0				
Component and disorders	0	1	2	3	4
General disorder, fatigue	49 (87.5)	7 (12.5)	0 (0)	0 (0)	
Cough	42 (75.0)	11 (19.6)	2 (3.6)	1 (1.8)	
Dyspnea	55 (98.2)	1 (1.8)	0 (0)	0 (0)	0 (0)
Pneumonitis	49 (87.5)	5 (8.9)	1 (1.8)	1 (1.8)	0(0)
Esophagitis	54 (96.4)	2 (3.6)	0 (0)	0 (0)	0 (0)
Rib fractures	56 (100)	0 (0)	0 (0)	0 (0)	0 (0)
Chest wall pain	53 (94.6)	3 (5.4)	0 (0)	0 (0)	0 (0)
Pleural and pericardial effusion	56 (100)	0 (0)	0 (0)	0 (0)	0 (0)

*Abbreviation: CTCAE* common terminology criteria adverse events

Data presented as number of treated patients, with percentages in parentheses

**Fig. 1:**
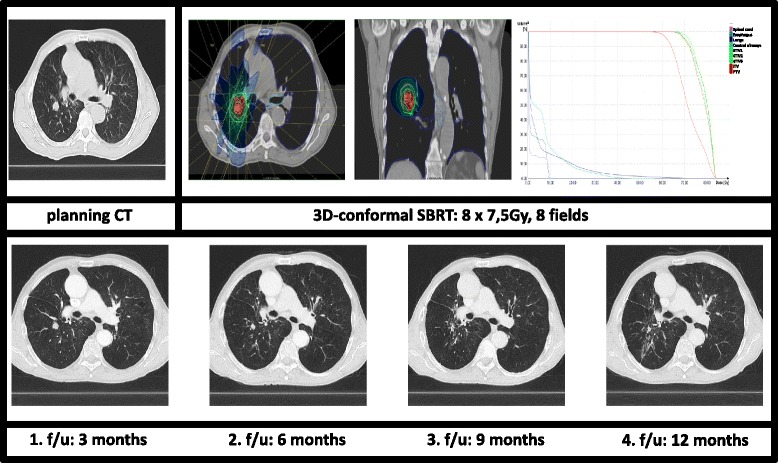
Treatment planning with dose-volume-histogram (PTV and OARs) and follow-up CT-scans for a central pulmonary tumor. Continuous tumor shrinkage with only rare signs of fibrosis

**Fig. 2:**
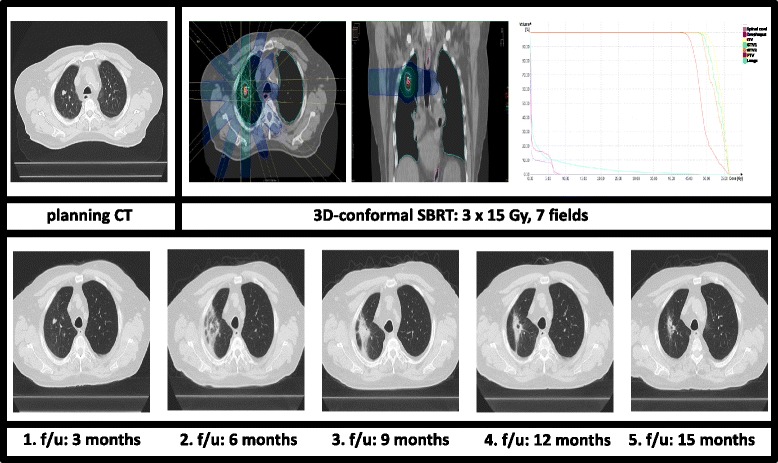
Treatment planning with dose-volume-histogram (PTV and OARs) and follow-up CT-scans for a peripheral pulmonary tumor. Radiation associated imaging changes: increasing perilesional, ground glass opacity leading to fibrosis

Only a subset of 43 patients (76.8 %) with a median follow-up time of 10.8 months (range 5.3–18.0 months) was available for late toxicity evaluation after 6 months. No toxicity greater than CTCAE 2° was reported (Table 4). However, radiation-induced pneumonitis rate increased by number but not by toxicity grade with 15 patients (34.9 %) suffering from pneumonitis CTCAE 1° and 3 patients (7 %) from CTCAE 2°. Figure 1 and 2 illustrate treatment planning and radiation-induced treatment changes for two exemplary patients.

**Table 4:** observed side-effects classified according to Common Terminology Criteria Adverse Events, version 4.0, during short-term follow-up (6 months after SBRT)

	CTCAE, version 4.0				
Component and disorders	0	1	2	3	4
General disorder, fatigue	43 (100)	0 (0)	0 (0)	0 (0)	
Cough	37(88.4)	4(9.3)	1(2.3)	0 (0)	
Dyspnea	42 (97.7)	1 (2.3)	0 (0)	0 (0)	0 (0)
Pneumonitis	25 (58.1)	15 (34.9)	3(7.0)	0(0)	
Esophagitis	42 (97.7)	1 (2.3)	0 (0)	0 (0)	0 (0)
Rib fractures	43 (100)	0 (0)	0 (0)	0 (0)	0 (0)
Chest wall pain	43 (100)	0 (0)	0 (0)	0 (0)	0 (0)
Pleural and pericardial effusion	43 (100)	0 (0)	0 (0)	0 (0)	0 (0)

*Abbreviation: CTCAE* common terminology criteria adverse events

Follow-up after 6 months was only available for 43 patients. Data presented as numbers of treated lesions, with percentages in parentheses

### Survival and local control

**Fig. 3:**
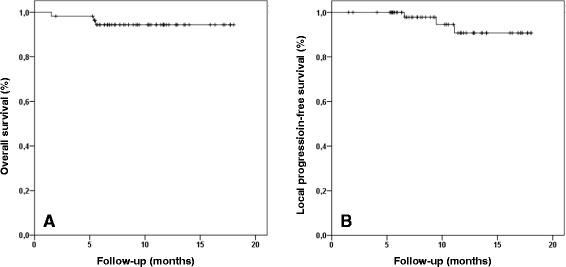
Overall survival (**a**) and local progression-free survival (**b**) after SBRT in FFF-mode

Median follow-up time was 9.3 months (range 1.5–18.0 months). 1-year OS and 1-year PFS were 94.4 % and 77.6 %, respectively. OS was significantly worse for patients with pulmonary metastases compared to primary NSCLC patients (*p* = 0.029). During follow-up time, only two patients developed local progression resulting in a 1-year LPFS of 92.8 % (Fig. 3). These two patients were also diagnosed with distant metastases at the same time. LPFS was not significantly different for primary NSCLC patients and patients with pulmonary metastases. Major failure pattern was distant with 1-year DPFS of 78.0 %. In total, 15 patients (26.8 %) suffered from distant progression during follow-up time. Patients with locally advanced NSCLC and patients with pulmonary metastases showed a strong tendency towards a higher risk of distant failure compared to early-stage NSCLC patients (*p* = 0.07). Distant relapses occurred in lungs (*n* = 9), brain (*n* = 4), liver (*n* = 2) and other organs (*n* = 4). Furthermore, OS, PFS as well as LPFS and DPFS were not significantly influenced by Karnofsky performance score, pulmonary function, toxicity, lesion size and localization (central vs. peripheral) as well as irradiation dose in biological effective dose (BED).

## Discussion

In the current study, SBRT treatment in FFF-mode was found to be effective and safe while reducing beam-on time to more than half compared to treatment in FF-mode.

Up to now, there are only few and heterogeneous studies investigating toxicity and outcome of SBRT with FFF-beams for lung tumors [21–24]. Most of these studies not only focus on SBRT for pulmonary lesions, but include various other tumors and locations treated with SBRT. Furthermore, applied doses and fractionation schemes are not homogeneous and often vary strongly. Hence, we report our initial experiences with the clinical implementation of SBRT using FFF-beams for 61 pulmonary lesions treated with homogeneous concepts.

Performing comparative plan analysis, we detected similar conformity indices for FFF-plans and FF-plans (*p* = 0.275). To our knowledge, there is only one further study which showed comparable plan quality for lung SBRT [25]. Furthermore, comparing treatment time for radiotherapy plans in FFF-mode to FF-plans in the respective technique, we detected a significant reduction. In detail, beam-on time was even reduced to more than half in median when comparing plans in FFF-mode to FF-technique. Others groups also confirmed accelerated dose delivery using the FFF-technique [21, 22, 26].

Although median follow-up time was rather short with 9.3 months, we detected 1-year local progression-free and overall survival rates of 92.8 % and 94.4 %, respectively. Recently, Stieb and colleagues described similar results investigating local progression-free and overall survival after SBRT treatment using FFF-techniques [24].

Biological concerns have been reported that SBRT applying FFF-beams might increase toxicity [27, 28]. There is an ongoing debate whether the applied dose rate directly affects cell survival and perhaps even toxicity. Recently, Lohse et al. demonstrated that irradiation of glioblastoma cell lines using FFF-beams was more efficient in reducing cell survival than the standard flattened beam. Additionally, this anti-tumor cell effect was more dependent on dose per pulse rate than on delivery time and became more evident with higher single doses [27]. Hence, increased toxicity after SBRT treatment in FFF-mode was discussed. However, Sorensen et al. and other groups did not detect any effect of instantaneous dose rate on cell survival applying high-dose rate FFF irradiation [29–31].

Despite the discussed altered radiobiology, we detected comparably low toxicity in our analysis. Only one patient (1.8 %) suffered from toxicity CTCAE 3°, while no toxicity CTCAE 4° and 5° were reported. Similar results were reported by Navarria et al. who analyzed 46 pulmonary lesions for early toxicity after SBRT applying FFF-beams [21]. There is only one study which reported comparably higher toxicity after SBRT in FFF-mode: Prendergast and colleagues described three patients with pulmonary toxicity CTCAE 3° and one patient each with CTCAE 4° and 5° pulmonary events analyzing 49 patients treated with SBRT for lung malignancies [28]. In this study, no comparison between FFF- and FF-mode was performed and higher biological effective doses were used in 29 % of the patients compared to our study [28]. In general, higher biological effective doses are expected to cause higher toxicity. Furthermore, doses and fractionation schemes are routinely adjusted to tumor location, as excessive toxicity after SBRT was reported for central tumors [32]. Hence, the different applied dose and fractionation schemes in the study of Pendergast et al. might explain the detected increased toxicity. Up to now, there is no convincing clinical evidence for the discussed increased toxicity for SBRT in FFF-mode. [deleted]

Despite the mentioned radiobiological concerns in respect to dose rate and toxicity, treatment applying FFF-plans is known to provide several dosimetric advantages compared to conventional unflattened photon beam therapy. Removing the flattening filter leads to a reduction of out-of field dose due to reduced head scatter, leaf transmission and lower dose outside the field edge [15, 16, 33]. Hence, sparing of the OARs should be more effective and lower toxicity rates are expected. Furthermore, Cashmore and colleagues even suggested that irradiation in FFF-mode might even reduce the risk of secondary malignancies by lowering doses by up to 70 % [34]. Limitations to our analysis were the relatively short follow-up time and the retrospective design of the study. Toxicity data was only available for the first 6 months for some patients and therefore later side-effects could only be evaluated for a subset of patients.

## Conclusions

In this retrospective analysis, we demonstrated that SBRT using FFF-techniques was time efficient while providing similar plan quality. Furthermore, early clinical results were promising with only modest toxicity.

## Abbreviations

BED: Biological effective dose
CBCT: Cone beam computed tomography
CC: Collapsed cone
CT: Computed tomography
CTCAE: Common terminology criteria of adverse events
CTV: Clinical tumor volume
DPFS: Distant progression-free survival
DRR: Digitally reconstructed radiographs
FDG-PET-CT: Fluoro-deoxy-glucose position emission tomography CT
FF: Flattening-filter
FFF: Flattening-filter free
GTV: Gross tumor volume
IMRT: Intensity-modulated radiotherapy
ITV: Internal target volume
LC: Local control
MV: Megavolt
NSCLC: Non-small cell lung cancer
OAR: Organs at risk
OS: Overall survival
PFS: Progression-free survival
PTV: Planning tumor volume
SBRT: Stereotactic body radiotherapy
VMAT: Volumetric modulated arc therapy
